# Distal Clavicle Osteochondroma Causing Supraspinatus Tendinopathy

**DOI:** 10.7759/cureus.4354

**Published:** 2019-04-01

**Authors:** Ioannis Galanopoulos, Panagiotis Stavlas, Michail Beltsios

**Affiliations:** 1 Orthopaedics, Thriasio General Hospital, Athens, GRC

**Keywords:** painful shoulder, supraspinatus tendinopathy, osteochondroma, distal clavicle

## Abstract

Shoulder pain is a very common symptom especially in young and active population. Rotator cuff tendinopathies are believed to be the most common cause of shoulder pain up to 86%. Tumors around the shoulder area can cause pain or joint stiffness when expanding in the subacromial space. We present a rare case of a distal clavicle osteochondroma. It is a benign tumor which in this area causes supraspinatus tendinopathy and every physician should suspect this kind of diseases during the diagnostic approach of a shoulder pain. In this case, early diagnosis and appropriate treatment with excision of the lesion gave us a very good outcome with fully relief of the symptoms.

## Introduction

The pain of the shoulder is a very common condition especially in young and active population. Except from the common diagnoses such as the impingement syndrome or traumatic lesions of the tendons and the labrum, less common causes can also exist [1]. The tumors of the shoulder area consist a rare group of diseases which should always be in surgeons' mind during the diagnostic approach. Tumors of the clavicle, benign and malignant, should be investigated.

## Case presentation

**Figure 1:**
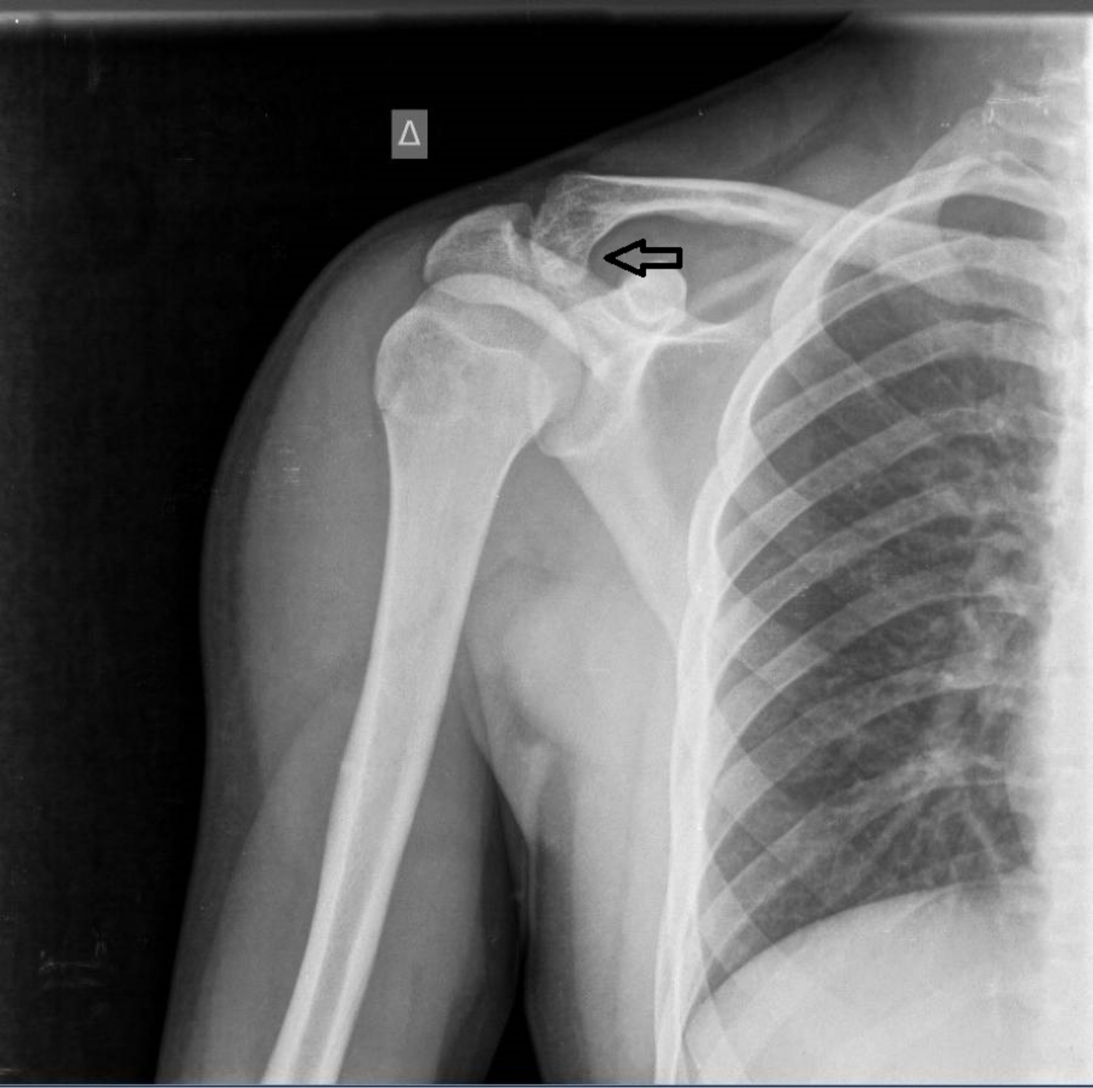
Anteroposterior radiograph of the right shoulder. A distal clavicle tumor is depicted.

A 17-year-old male presented with persistent pain of the right shoulder for two months. The pain was exacerbated during abduction and external rotation of the shoulder. The clinical examination was consisted with supraspinatus tendinopathy. The plain radiographs of the right shoulder revealed a solitary lesion of the distal clavicle suggestive for a benign bone lesion (Figure 1).

**Figure 2:**
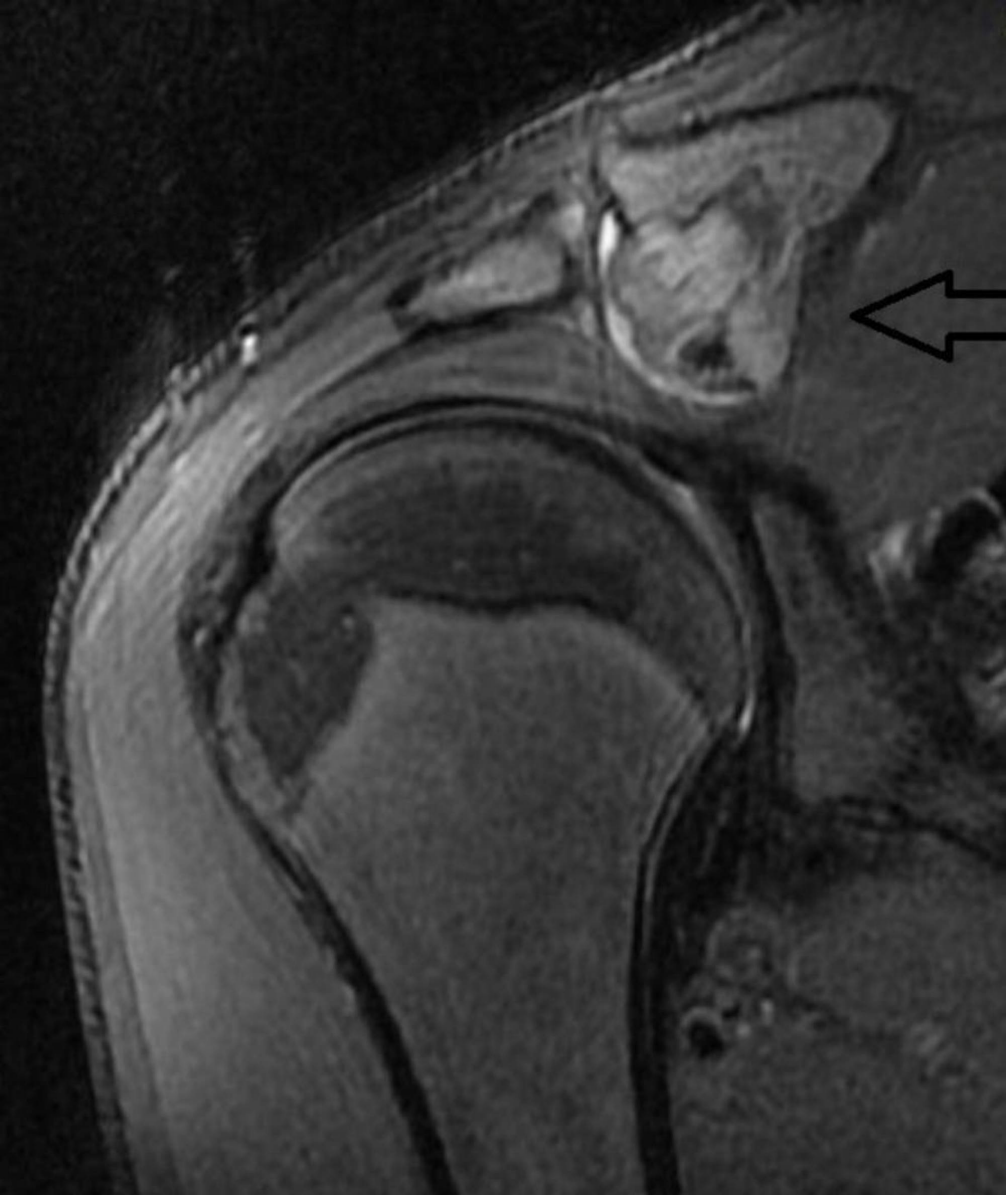
Magnetic resonance imaging (MRI) of the lesion highlights benign characteristics.

The magnetic resonance imaging (MRI) was consisted with a distal clavicle tumor with benign characteristics arising from the distal posterior-inferior surface of the bone (Figure 2).

**Figure 3:**
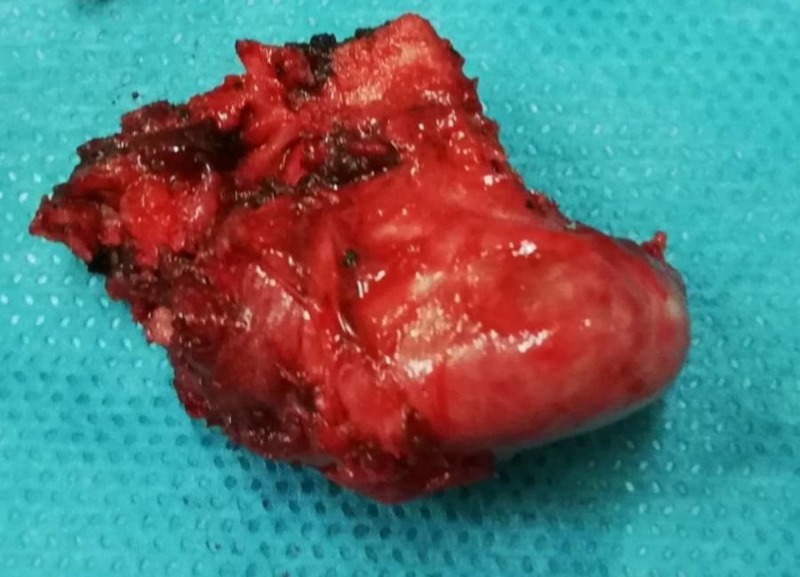
The tumor has been fully excised.

The mass seemed to limit the available space for the supraspinatus muscle near to the musculotendinous junction. An open resection of the tumor was scheduled. With the patient in beach chair position, a longitudinal incision between the distal clavicle and the coracoid process was performed followed by superficial and deep dissection of the soft tissues. The distal clavicle was palpated and a subperiostical exposure of the anterior surface was performed. Because of the posterior-inferior location of the tumor, a distal clavicle resection about one centimeter (cm) from the acromioclavicular (AC) joint was necessary for better approach. With the use of a saw and a curved osteotome the tumor was completely resected including the adjacent clavicle cortex (Figure 3).

**Figure 4:**
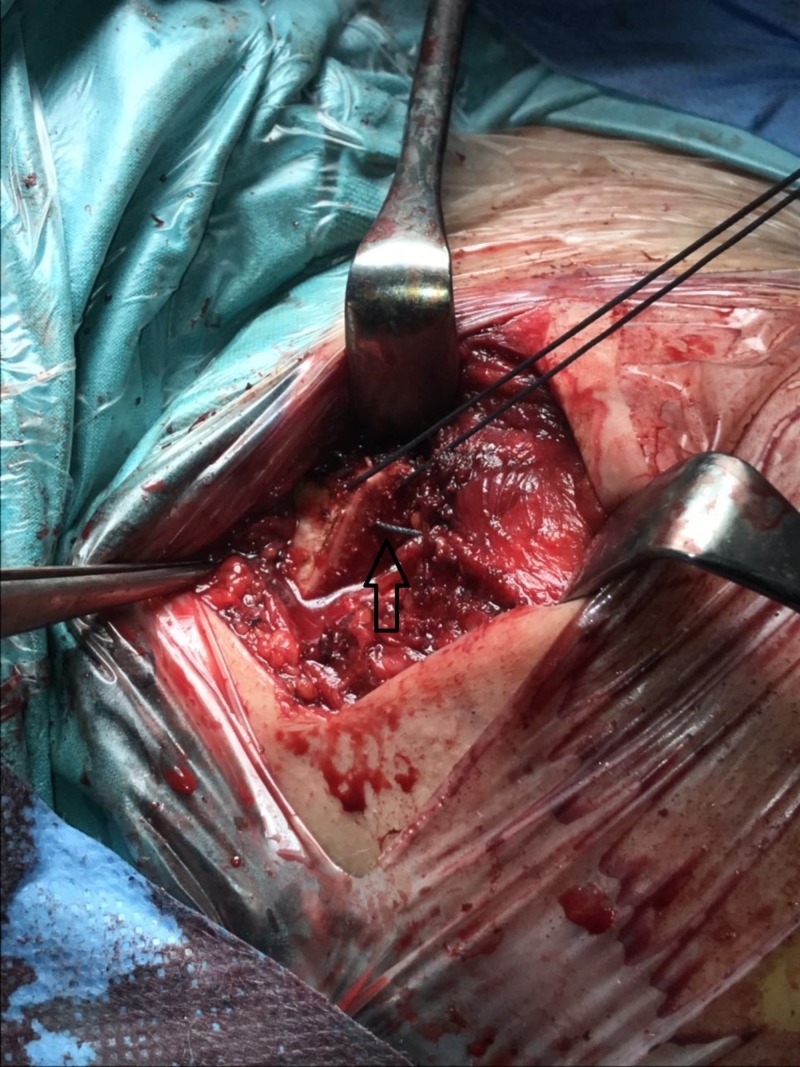
Transosseous sutures for the reattachment of the anterior deltoid muscle to the clavicle bone.

The coracoclavicular ligaments were checked intraoperatively and found intact. However, an anterior deltoid reattachment to the distal clavicle, through transosseous nonabsorbable sutures, was performed to secure the stability of the clavicle (Figure 4).

**Figure 5:**
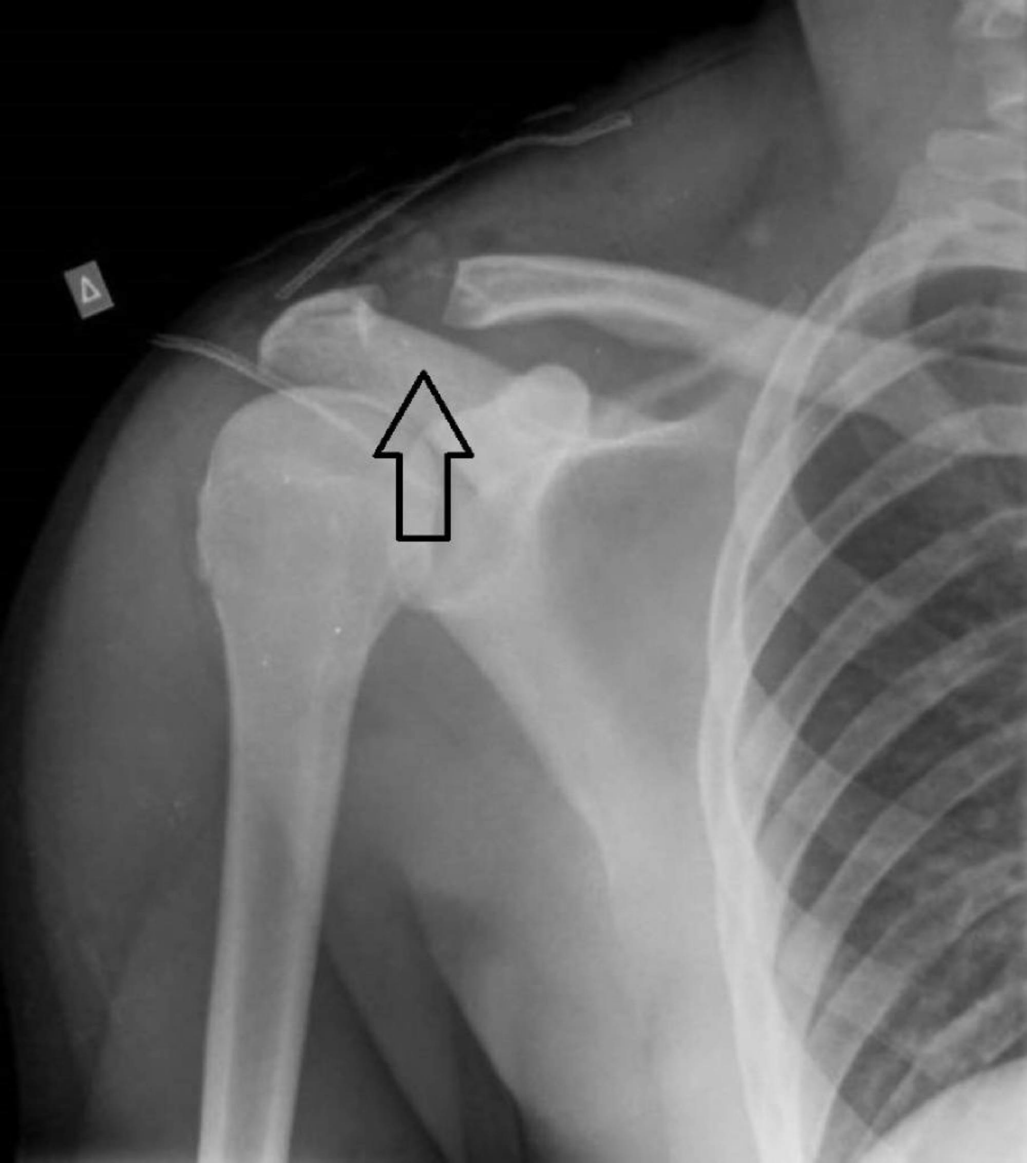
Postoperative anteroposterior radiograph. The tumor is fully excised.

The postoperative radiograph showed complete resection of the tumor (Figure 5).

The arm was protected in sling immobilization for three weeks. The pathology report confirmed the diagnosis of osteochondroma. Gentle exercises were permitted after three weeks with full range of motion (ROM). In six weeks postoperatively, the patient returned to his normal activities and 10 weeks postoperatively he got back in sport activities.

## Discussion

Osteochondroma is a true benign bone tumor arising on the external surface of a bone, containing a cavity of marrow covered by cartilage. It is the second most common benign bone tumor following nonossifying fibroma formed by three layers-bone, cartilage and perichondrium [2]. The cartilage cap is thin, less than 2 mm thick, and the close to the bone cartilage is similar to the growth plate cells. These cells are organized into chords which undergo enchondral ossification. An osteochondroma appears as a stalk or a flat protuberance emerging from the surface of the bone or it ends up as a hook-like formation. It is usually presented on the metaphyses and the insertion points of tendons and as a result, the metaphysis near the lesion can be widened. An osteochondroma has usually smooth margins and rarely irregular [3]. Atypical characteristics of these lesions may indicate malignant transformation although it is a really rare phenomenon [4, 5]. Eighty-five percent of osteochondromas are presented as solitary lesions and 15% as multiple exostosis. Children and adolescents are the most common patients suffered by osteochondromas which are slowing growth lesions. Depending on the location of an osteochondroma, various symptoms may develop such as bony deformities, mechanical joint problems, fractures or impingement syndromes when the lesion is near to tendons or muscles. Vascular or neurologic compromise could also be present [6, 7].

Benign and malignant tumors of the clavicle are not common although currently, clavicle bone tumors are more commonly recognized than in the past [8-10]. Osteochondroma represents 8.7% of the clavicle tumors. The lateral third of the clavicle is affected by bone tumors in 33.6% [11].

The reports about distal clavicle osteochondromas are limited in the literature. Ogawa et al. reported two cases with painful shoulder because of impingement syndrome caused by a distal clavicle osteochondroma [12]. Vander Maren et al. reported a case of a 47-year-old man with rotator cuff tendinitis because of an osteochondroma of the coracoclavicular area. The authors chose the deltopectoral approach to excise the tumor and they also highlight the function of the cartilage of this area as a growth plate [13]. Reichmister et al. reported two cases of distal clavicle osteochondroma with complete relief of the symptoms after the tumor resection [14].

A painful shoulder with rotator cuff tendinitis symptoms should be suspicious for distal clavicle osteochondroma. Although this lesion is not common, delayed diagnosis could be harmful for the patient. A delayed diagnosis of such a case seven years after the beginning of the symptoms is reported in the literature [15].

In our case we preferred an open excision in order the tumor to be excised in clear margins although the imaging characteristics of the tumor were suggested for a benign lesion. However, an arthroscopic excision has been described in 2011 [16]. No recurrence has been reported in recent studies [17]. Malignant transformation of osteochondromas is lower than 1% according to the literature [18].

In diagnostic approach, MRI imaging seems to be very useful and highlights the cartilage tissue of the lesion [19]. Preoperative needle biopsy for these lesions does not seem to be familiar among orthopaedic surgeons although some authors suggest biopsy in case of diagnostic controversy [20].

## Conclusions

Distal clavicle osteochondroma is not common. However, it can present and cause shoulder pain and stiffness because of subacromial space narrowing and supraspinatus tendinopathy. Early diagnosis and treatment is necessary for a good outcome.
